# Supplementary material for: Small pangenome of Candida parapsilosis reflects overall low intraspecific diversity
Source: mBio. 2025 Aug 25;16(10):e01320-25. doi: 10.1128/mbio.01320-25 (PMC12505961; doi:10.1128/mbio.01320-25)
Supplement: Supplemental Figures — Figures S1 to S6. [file mbio.01320-25-s0001.pdf]

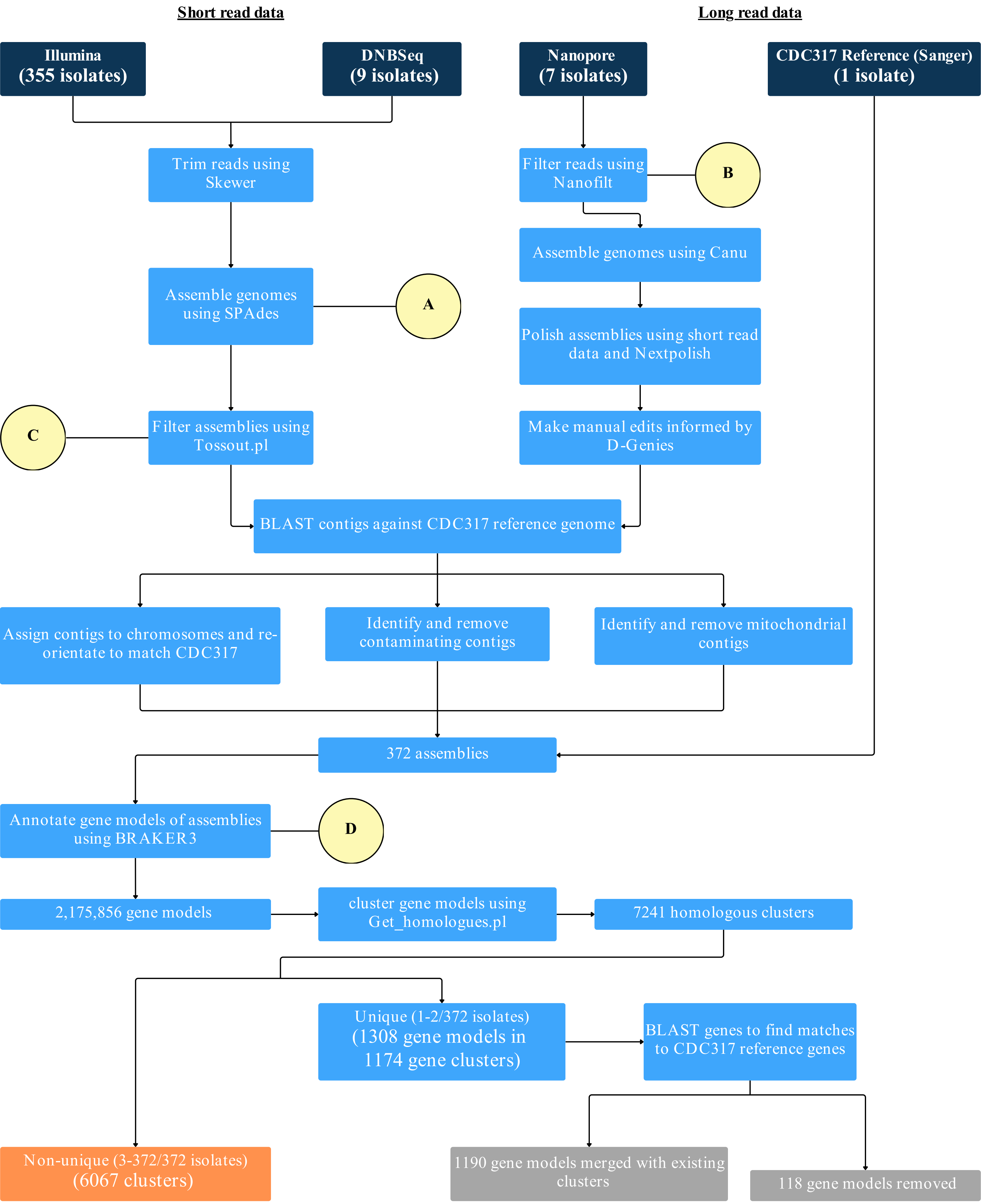

Supplementary Figure 1. Genome assembly, annotation and gene clustering pipeline

Supplementary Figure 1. Genome assembly, annotation and gene clustering pipeline. Pipeline shows software and steps taken to assemble, annotate and filter isolate genomes, and perform clustering. Steps taken are indicated by arrows and boxes in dark blue, blue, orange and grey. Steps in dark blue are the beginning of the pipeline, steps in blue are the middle of the pipeline, steps in grey are completed and not analysed further, and steps in orange are continued in Supplementary Figure 2. Yellow circles with letters indicate configuration settings of software as described in the methods: (A) minimum mean read lengths of 35 and minimum mean read qualities of 30; (B) minimum read quality of 7 and minimum read length of 1 kb; (C) minimum contig 113 coverage of 10 and minimum contig length of 500 b; (D) Train using orthogroups from CGOB with presence in three or more species, and set intron_downsampling to 0.

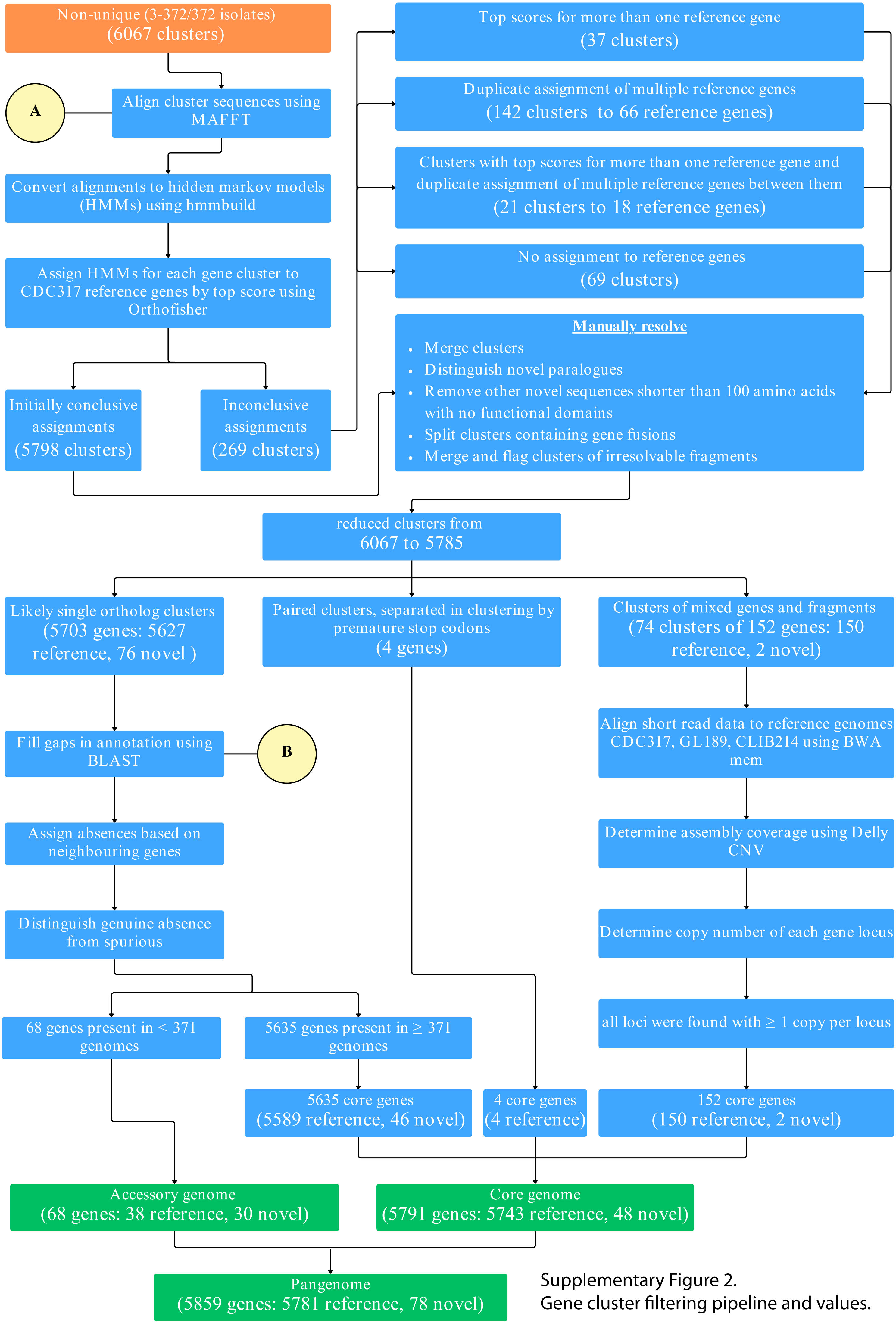

Supplementary Figure 2.
Gene cluster filtering pipeline and values.

Supplementary Figure 2. Gene cluster filtering pipeline and values. Pipeline shows software and steps taken to filter gene clusters and determine presence absence. Steps taken are indicated by arrows and boxes in orange, blue and green. Steps in orange are continuing from Supplementary Figure 1, steps in blue are the middle of the pipeline, steps in green represent the end result of the pipeline. Yellow circles with letters indicate configuration 115 settings of software as described in the methods: (A) Run MAFFT with parameters "--maxiterate 1000 --op 1.0 –genafpair"; (B) Filter hits by 99% identity and 99% coverage length.

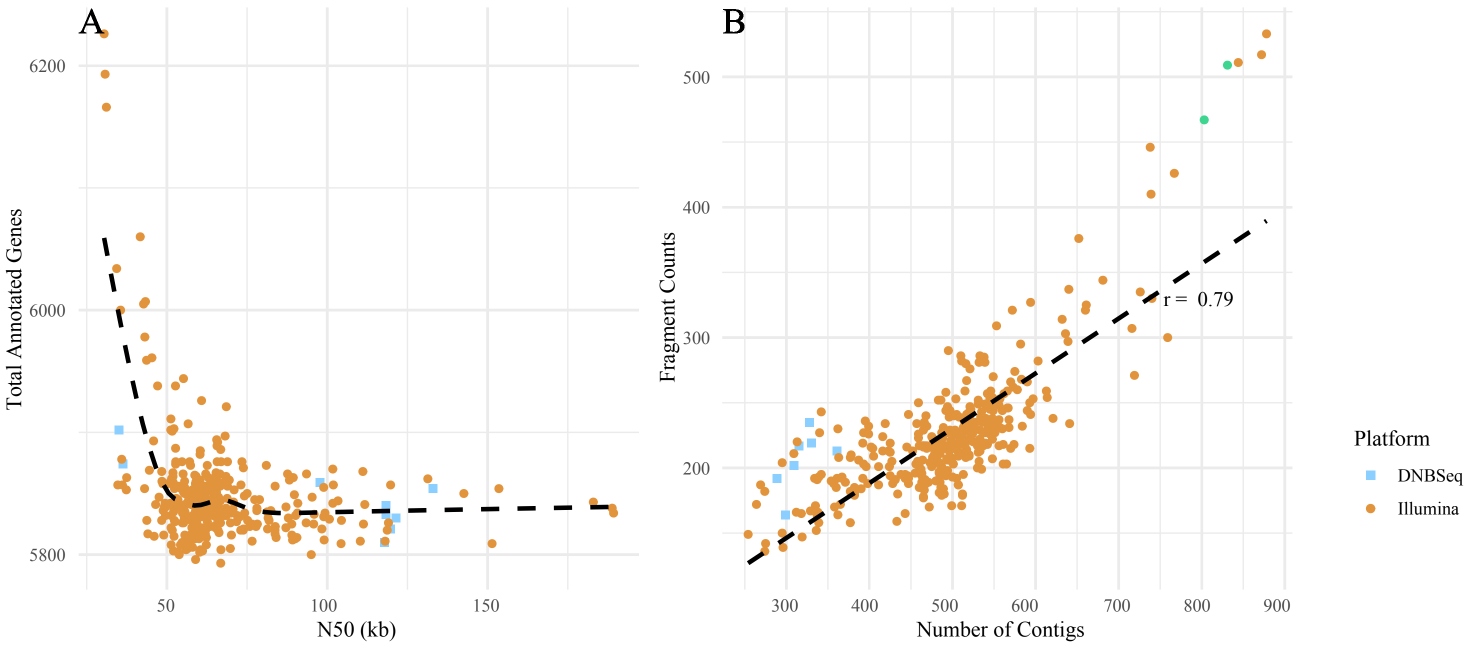

Supplementary Figure 3. (A) Scatter plot of N50 values versus annotated gene counts annotated by BRAKER3. Each dot corresponds to the N50 value (X-axis) in kilobases and annotated gene count (Y-axis) per sample. A regression under gamma parameters is depicted by the black dotted line. Samples are coloured by sequencing platform, as indicated; (B) Scatter plot of contig counts (X-axis) versus fragmented gene counts (Y-axis) per sample. A linear regression is depicted by the black dotted line. A Pearson correlation of 0.79 was observed. Colouring as in (A). For clarity, only scaffold-level assemblies are shown.

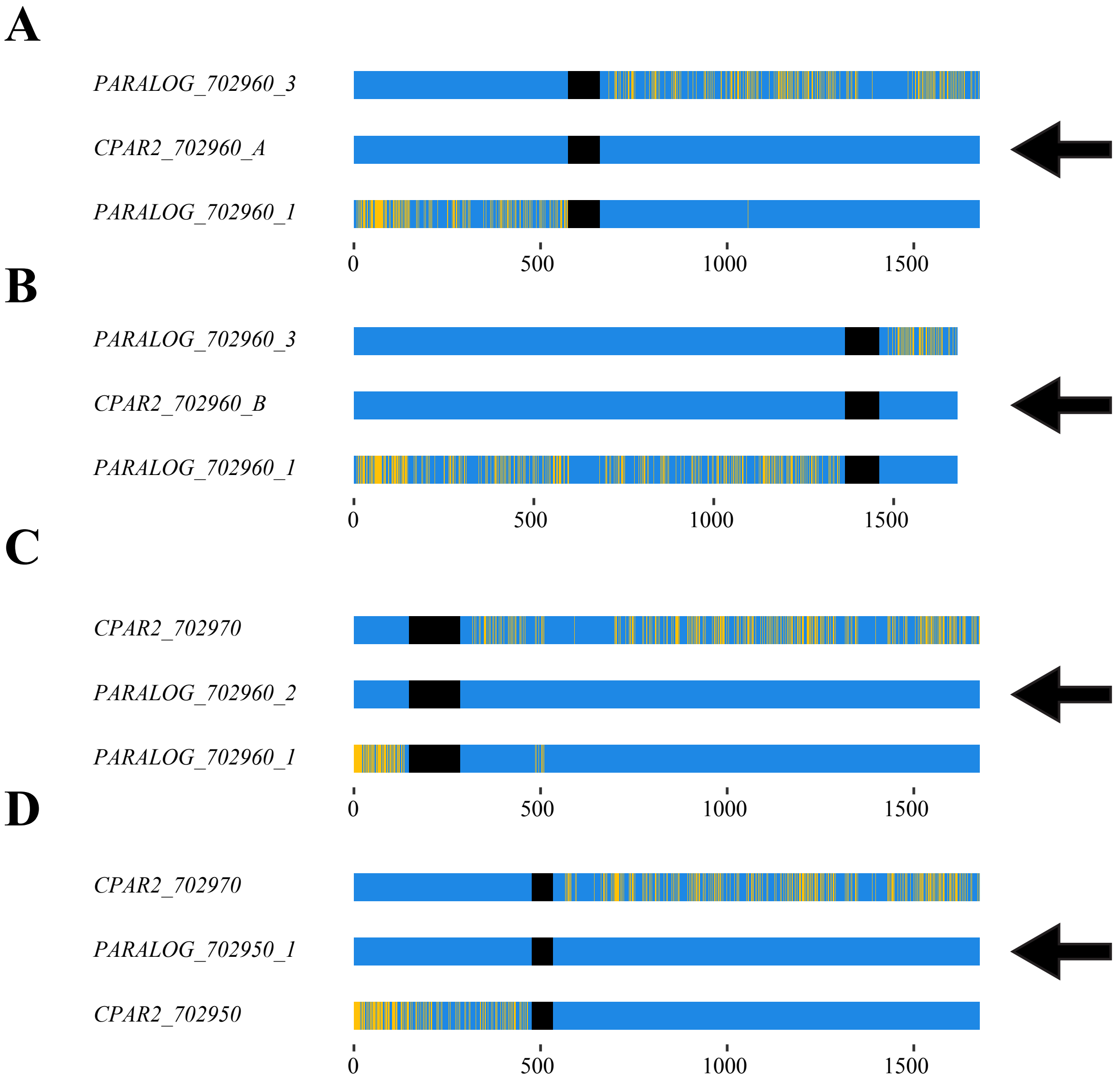

Supplementary Figure 4. Structure of SOA1 homolog fusions. CLUSTALW alignment of SOA1 homologs visualised using R package seqvisr (Vragh, 2022). Sites matching the fusion gene sequences (indicated by the arrow) are shown in blue and mismatches in yellow. Black bands indicate shared identical regions between fused 5' and 3' regions between parental sequences. White bands indicate gaps in the alignment. Sequence names are as indicated, fusion genes are further highlighted by arrows to the right. (A) Alignment of PARALOG_702960_1 and PARALOG_702960_3 with Type 2A fusion gene CPAR2_702960_A. Nucleotides 1 to 573 are identical PARALOG_702960_3, nucleotides 574 to 659 are identical to both PARALOG_702960_1 and PARALOG_702960_3, and nucleotides 660 to 1653 are identical to PARALOG_702960_1 with one site of difference. (B) Alignment of PARALOG_702960_1 and PARALOG_702960_3 with Type 2B fusion gene CPAR2_702960_B. Nucleotides 1 to 1365 are identical to PARALOG_702960_3, nucleotides 1366 to 1460 are identical to both PARALOG_702960_1 and PARALOG_702960_3, and nucleotides 1461 to 1653 are identical to PARALOG_702960_1. (C) Alignment of PARALOG_702960_1 and CPAR2_702970 with related Type 3 fusion gene PARALOG_702960_2. Nucleotides 1 to 147 of PARALOG_702960_2 are identical to CPAR2_702970, nucleotides 148 to 284 nucleotides are identical to PARALOG_702960_1, nucleotides 427 to 557 are identical to 119 both PARALOG_702960_1 and CPAR2_702970 with 8 sites of difference from either parent, and nucleotides 558 to 1644 are identical to PARALOG_702960_1. (D) Alignment of CPAR2_702950 and CPAR2_702970 with related Type 4 fusion gene PARALOG_702950_1. Nucleotides 1 to 477 of PARALOG_702950_1 are identical to CPAR2_702970, 478 to 533 are identical in all three genes, and positions 534 to 1644 are identical to CPAR2_702950.

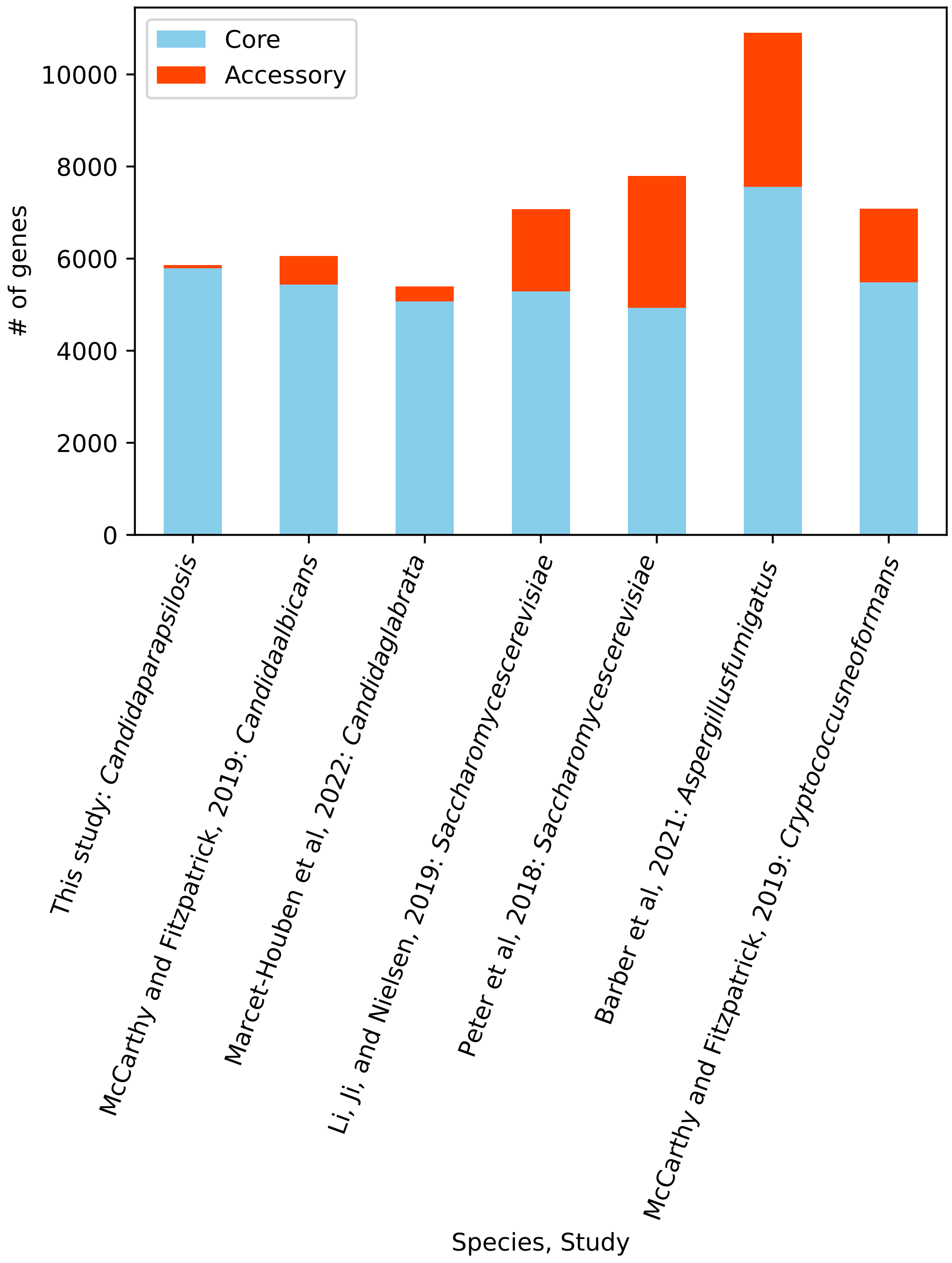

Supplementary Figure 5. Comparison between pangenome sizes of different fungal species. Each bar represents a separate species' pangenome from different studies. The cyan portion shows the number of core genes and red shows the number of identified genes not present in the core genome. This includes accessory genes and unique genes.

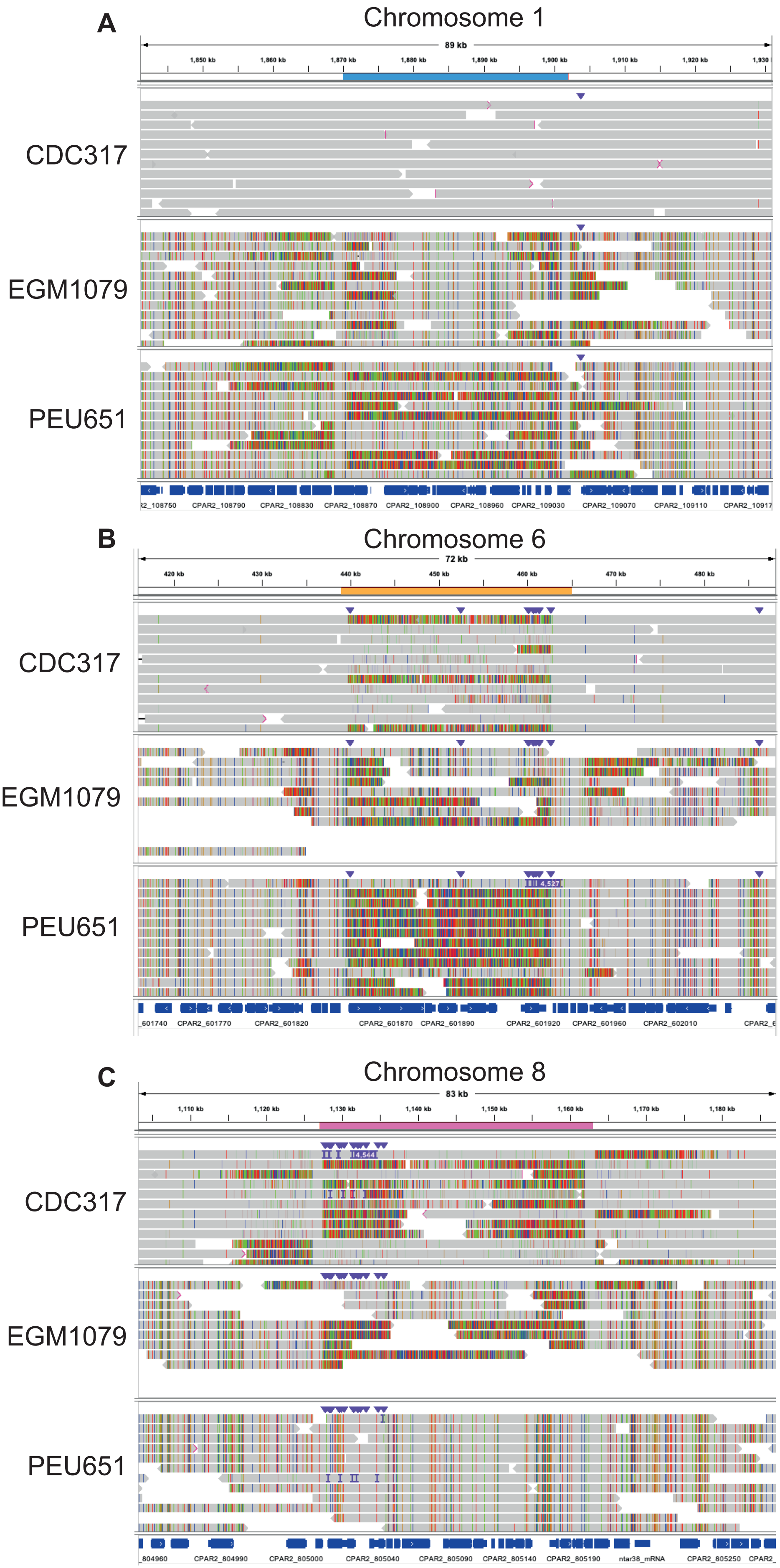

Supplementary Figure 6. Alignment of long reads at inversion sites.
IGV screenshots showing alignment of long reads for strains CDC317, EGM1079, and PEU651 against the CDC317 reference genome at sites of three inversions. Grey bars show individual reads mapped to the reference. Colored lines indicate mismatches between the read and the reference. Indels shorter than 30bp have been removed for visual clarity. Colored bars at the top of each diagram show the breakpoints of the inversions and correspond to colors in Figure 6.
